# Type 1 diabetes through two lenses: comparing adolescent and parental perspectives with photovoice

**DOI:** 10.1186/s13633-016-0020-z

**Published:** 2016-01-20

**Authors:** Ashby Walker, Desmond Schatz, Cathryn Johnson, Janet Silverstein, Shannon Lyles, Henry Rohrs

**Affiliations:** The Department of Health Outcomes and Policy and the Institute for Child Health Policy, University of Florida, PO Box 100177, Gainesville, 32610-0177 FL USA; The Department of Pediatrics, University of Florida, PO Box 100296, Gainesville, FL 32610-0296 USA; The Department of Sociology, Emory University, 1555 Dickey Drive, Atlanta, GA 30322 USA

**Keywords:** Type 1 diabetes, Adolescents, Glycemic control, Parents, Caregivers, Photovoice

## Abstract

**Background:**

Parental support and care-coordination are vital for youth with type 1 diabetes (T1D) in achieving positive health outcomes. Yet, studies are rarely designed to identify factors that influence parent/youth collaboration or how their perspectives about diabetes may vary.

**Methods:**

Photovoice was used to explore how adolescent and parental perspectives on T1D compare to identify factors that may influence care collaboration. A follow-up study was conducted where parents/caregivers of adolescents with T1D were prompted to take and explain five photos capturing what diabetes meant to them. Selection criteria included having a child 12–19 years with a diagnosis of T1D (≥2 years since onset). Thirty-three parents/caregivers participated (24 mothers, six fathers, two grandmothers, and one grandfather of 19 sons/14 daughters; mean age 15 years [±2.1]; mean disease duration 6 years [±3.3]). Content analysis was used to compare parent/caregiver photos with those captured by adolescents in a previous study with 40 youth participants (20 males/20 females; mean age 15 years [±1.9]; mean disease duration 6 years [±3.9]) through a method of constant comparison. Socioeconomic status was measured by household income and parental education. Glycemic control was captured by HbA1c. Mann-Whitney U testing was used to compare representations across demographic variables (202 youth photos, 153 parental photos).

**Results:**

Over half of adolescents and parents took at least one photo of: (1) diabetes supplies (2) food (3) coping mechanisms/resilience and (4) disease encroachment. Parents and adolescents similarly framed food-related issues as a major source of frustration in diabetes care. However, narratives about diabetes supplies differed: adolescents framed supplies as a negative aspect of diabetes whereas parents tended to celebrate supplies as improving life. Also, images of disease encroachment differed: adolescents took photos of their bodies to depict how diabetes trespasses on their lives whereas parents took pictures of clocks to denote sleep disruption or exhaustion from constant care demands.

**Conclusions:**

Food-related issues and varying views on supplies may trigger diabetes-specific conflicts. Contrasting viewpoints about the most cumbersome aspects of diabetes may provide insight into differential paths for interventions aimed at offsetting the burdens of T1D for adolescents and parents.

## Background

Navigating the demands of type 1 diabetes (T1D) can be particularly challenging during adolescence. Studies consistently show that maintaining near optimal glycemic control is difficult during this time period due to rapid physiological as well as psychosocial changes that accompany the transition to adulthood [1–3]. Amidst these challenges, high levels of care coordination between parents and youth are required to achieve targets for glycemic control. Parental involvement, support, and consistent monitoring of diabetes care improve glycemic control for adolescents living with T1D [4–7]. Conversely, diabetes-specific family conflict is identified as one of the greatest barriers to achieving optimal glycemic control in T1D for adolescents [8–12].

It is vital to identify potential triggers or causes of diabetes-specific conflict for families living with T1D given its established importance in glycemic control. It is certainly understood that diabetes, itself, creates tremendous strain on the ecosystem of a family unit with intensive insulin regimens, constant food mindfulness, and the irrevocability of living with a chronic illness. However, pinpointing specific areas related to diabetes experiences within families that breed conflict is necessary to develop possible solutions.

To better understand basic perspectives on the ways adolescents living with T1D and their parents/caregivers perceive and experience this disease, we conducted an exploratory study using photovoice [13]. Photovoice is a method where participants are asked to frame some part of their lived experience or lives through the use of photography [13–17]. The methodology from a previous study we conducted with adolescents [18–20] was replicated by providing disposable cameras to parents/caregivers of adolescents living with T1D and prompting them to *“take five pictures of what diabetes means to you.”* A systematic content analysis was conducted of the parental/caregiver photo narratives and compared to previously collected adolescent photo narratives to identify similarities and differences among representations. Adolescents and parents similarly framed food-related issues as a source of frustration in diabetes care, but demonstrated fundamentally different perspectives regarding diabetes supplies and themes of disease encroachment.

## Methods

Recruitment for this research took place in routine pediatric endocrine clinics at a large university hospital and its affiliated diabetes camps. The study protocol was approved by the IRB-01 (University of Florida) at the institution where the research took place and included informed consent and assent for study participants. In the first study, youth between the ages of 12–19 years who had a diagnosis of T1D of two years or more since onset were enrolled. Participation involved a brief youth survey as well as a survey for parents to complete that addressed basic questions about living with T1D as well as demographic information. In addition to the brief surveys, youth participating in the research were provided with a disposable camera and given the following directions:

*You know better than anyone what it is like to live with diabetes — and your involvement in this study will allow us to better understand your experiences. With the disposable camera you have been provided, take 5 pictures that show what diabetes means to you. As you take each picture, keep the following idea in mind: To me, diabetes is ____. Imagine explaining what it means to live with diabetes to someone who has no idea what that may be like. For each picture you take, make sure to complete the table on the next page to help explain your choices.*

The table contained in the survey provided a space for participants to list the picture taken (“list the pictures taken”) and then provide a narrative explanation of what that particular picture represented to them about diabetes (“explain what each picture represents about diabetes to you.”) All research survey materials and disposable cameras were taken home for completion and youth participants were provided with a postage-paid envelope to return them. A $30 money order was sent to youth when they returned their materials. In an effort to ensure high return rates, reminder postcards were mailed after 2 weeks, reminder phone calls made after 1 month, and then an entire new packet of materials mailed to non-responders after 2 months.

A year following the completion of the first study we replicated these same procedures in a follow-up study with parents/caregivers of T1D adolescents who met the same selection criteria. Parents/caregivers were given the exact same instructions with provided disposable cameras. In cases where an adult other than a parent had full custody, was legal guardian for a youth, or lived in the same household with the youth, they were eligible to participate. Parents who had a youth participate and return completed materials in the first study were all mailed recruitment packets with a letter explaining the new study and inviting them to participate. New enrollment also took place in clinics and related diabetes camps with parents and caregivers. A $10 Starbucks card was sent to each parent/caregiver who participated in the second study.

Several demographic measures were captured in surveys completed by the parents. Socioeconomic status (SES) was measured several ways including total household income and parental education (both in years and by degree type). Other demographic variables included gender, age (for youth, parents, and all persons within the household), race/ethnicity, years of disease duration, and marital status. HbA1c was also recorded from patient records as an indicator of glycemic control.

Data analysis was multilayered and mimicked techniques utilized in the first study with adolescents. A content analysis of photos and photo narratives was conducted to determine the major categories of representation presented by parents/caregivers. This analysis involved a hybrid technique of quantitative content analysis [21, 22] as well as a qualitative method of constant comparison associated with grounded theory [23, 24] to determine major themes of representation. Thus, the analysis involved an interplay of quantitative content analysis whereby the number of photos was counted according to a typology that was developed by multiple coders (“what” coding – x number of pictures were of diabetes supplies, x number of photos were of food, and so on) and subsequently refining and systematizing these categories through qualitative comparison of the narrative meanings provided for them (“why” coding -- x% of the supplies photos represented challenges, x% of the supplies photos represented describing their use, etc.). Photo-index scores [18–20] were subsequently developed to indicate the total number of pictures taken by a given parent/caregiver for each of the major photo categories (e.g. a photo-index score of two meant that a parent devoted two of her five pictures to food depictions).

All quantitative measures were analyzed using SPSS (Statistical Package for the Social Sciences; Version 21 when the research began but Version 22 by its completion – IBM, Armonk, NY), while atlas.ti7 (GmbH, Berlin) served as the workbench for qualitative analysis. Multiple coders participated to establish adequate levels of inter-coder reliability [25] (>95 % for youth photos and >90 % for parent photos). Pearson’s correlation was used to examine relationships among our quantitative measures like SES (income) and HbA1c, and photo-index scores were compared among different groupings within our sample using Mann-Whitney U testing for statistical significance.

## Results and discussion

The overall demographic characteristics of our sample are presented in Table 1. Forty youth and 33 parents/caregivers completed and returned photo projects. Roughly half of the individuals enrolled in the study returned completed packets (a 50 % return rate for both studies); however, the demographics of the participants who completed and returned packets mirrored the larger overall enrollment sample, as well as the demographic composition of the clinics where recruitment took place. In both samples the mean age of youth was 15 years. The mean disease duration was 6 years, with relatively equal gender ratios and an overrepresentation of white participants. Parent/caregiver surveys were completed by 24 mothers, six fathers, two grandmothers, and one grandfather. There were eight parent/youth dyads where both a youth and parent/caregiver returned packets from the same family, five mother/father dyads, and 1 grandmother/grandfather dyad – with a total of 58 distinct households participating in this project (two families had sibling pairs). A range of SES households were also represented in both samples (see Table 1). SES was a statistically significant predictor of glycemic control when HbA1c was correlated with household income (*r* = −.47, *p* < 0.05) or years of parental education (*r* = −.64, *p* < 0.01).

**Table 1:** Sample Characteristics

Youth Sample			Parent/Caregiver Sample		
	*n* = 40	HbA1c		*n* = 33	HbA1c
			*Completed By: 24 mothers, 5 fathers, 1 stepfather, 2 grandmothers, 1 grandfather*		
Age	15 ± 1.9		Age	15 ± 2.1	
T1D Duration	6 ± 3.9		T1D Duration	6 ± 3.3	
Gender			Gender		
Female	20	8.5 % ± 0.8	Daughters	14	8.3 % ± 1.3
Male	20	8.6 % ± 1.5	Sons	19	8.2 % ± 1.6
Race/ethnicity			Race/ethnicity		
Black	3	10.2 % ± 0.6	Black	3	8.7 % ± 3.0
Hispanic	3	8.8 % ± 0.4	Hispanic	1	10 %
White	33	8.5 % ± 1.2 --	White	26	8.1 % ± 1.3
Other	1	--	Missing Data	3	----
SES			SES		
< $40,000	13	9.5 % ± 1.6	< $40,000	8	9.3 % ± 1.9
$40,000-$80,000	14	8.2 % ± 0.9	$40,000-$80,000	9	7.9 % ± 0.9
>$80,000	12	8.3 % ± 0.6	>$80,000	13	7.5 % ± 0.7
Missing Data	1	----	Missing Data	3	----

**Fig. 1:**
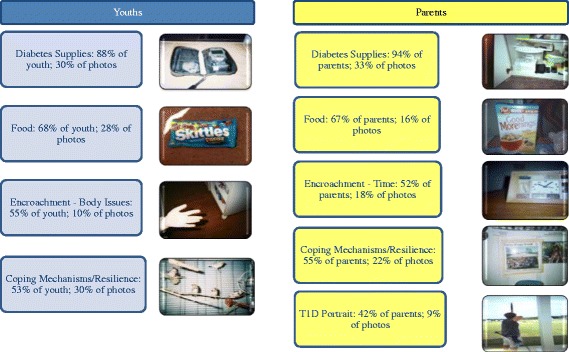
Content Analysis of Photos. Youth: *(n* *= 40* *)*, 202 photos; Parents: *(n* *= 33* *)*, 153 photos

Findings from the overall content analysis of photos are presented in Figure 1. The 40 adolescents participating in this research collectively took 202 photos and the 33 parents took 153 photos. In both samples, there were a few individuals who took slightly more or less than the requested five photos each, thus totaling 355 photo narratives for analysis. For parents/caregivers and youth, 50 % took at least one photo of the following types of representations: (1) diabetes supplies, (2) food as a source of frustration, (3) encroachment (images that depict where diabetes infringes on their lives), and (4) coping mechanisms/resilience (activities that help mitigate the challenges of diabetes or images that show active resistance to negative disease associations). One additional type of major representation was unique to the parents/caregivers: a T1D portrait that involved taking a picture of their child who lives with T1D. These pictures were accompanied by narratives that conveyed the pain they felt for not being able to take this diagnosis away for their children, yet also words that described tremendous pride in how their beloved were dealing with T1D.

## Areas of similarity: *food as frustration*

For families living with T1D in this sample, food-related issues are universally identified as a source of frustration and it is openly acknowledged as a source of diabetes-specific conflict within homes (Table 2). Youth captured images of candy, carbs, and sugary beverages to denote foods they feel they have to limit or negotiate with parents continuously. A 14-year-old male took a picture of a bag of Skittles with the narrative: *“You want candy? TOO BAD. You have diabetes.”* Youth also simultaneously conveyed disdain for the public’s misunderstanding of diabetes and the tendency for others to lecture them about what was perceived to be off-limits. A 15-year-old male said *“I WANT THEM (public) TO KNOW THAT WE CAN EAT SUGAR AND NOT DIE HORRIBLY!”* A father of a 17-year-old female described encounters over food intake choices as a “battleground” in his household. The majority of mothers took pictures of nutrition labels or scales to show the hard work that accompanies nutrition decisions, and narratives of guilt commonly accompanied these pictures. They expressed fear/worry about miscounting carbs but also guilt over the ways food-mindfulness for the child living with diabetes impacted siblings who did not have diabetes (see Table 2 for examples).

**Table 2:**
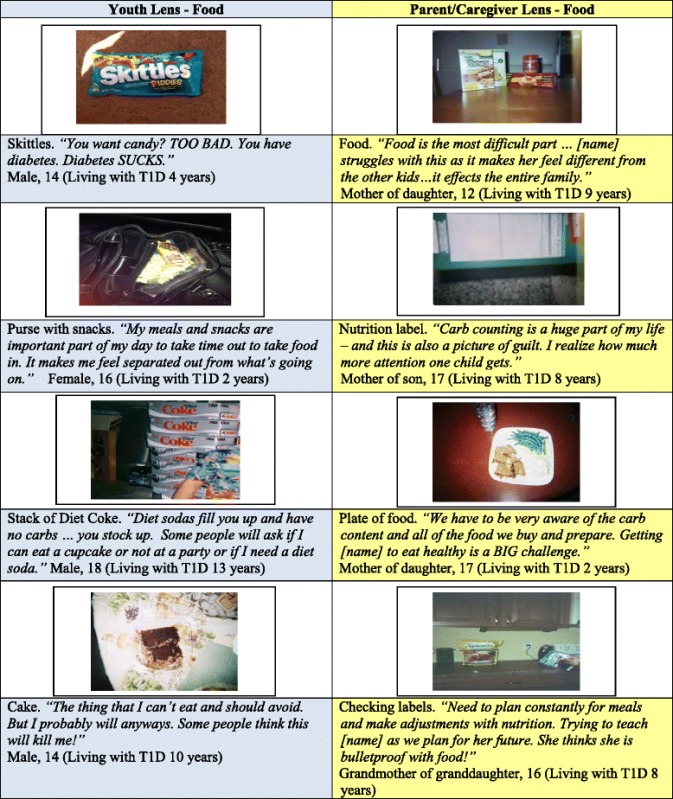
Areas of similarity: *Photos of food*

## Areas of contrast: *diabetes supplies and encroachment*

For all participants, photos of diabetes supplies (needles, insulin, test strips, pumps, pens etc.) were the most common representation of *“what diabetes means.”* However, youth and parents/caregivers had different narratives surrounding supplies photos. For youth, supplies like insulin and pumps are shown as something they can never get away from and that constantly identifies them to the world as someone with T1D (Table 3). These supplies are shown as taking over much space within their homes *(“MANY drawers in my house are devoted to supplies,”* a 19-year-old female says) and requiring a presence in every space of their lives (*“You have to take your medicine…wherever you are,”* a 17-year-old female says about a picture of her insulin at a restaurant). Moreover, the supplies call them out publically as “different” and often evoke unwanted questions from others around them (Table 3).

**Table 3:**
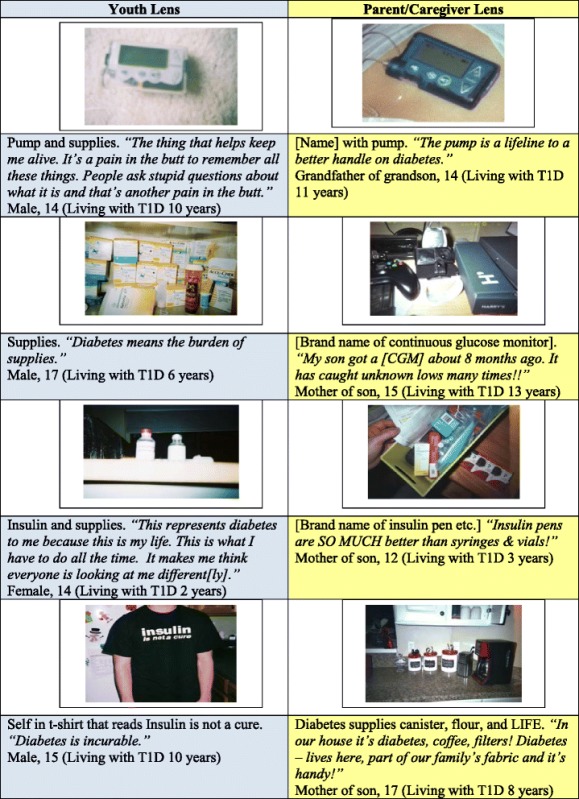
Areas of contrast: *Photos of supplies*

Youth depicted supplies as *limiting* their freedom; parents, however, often described supplies and specific technologies related to insulin and blood glucose monitoring (BGM) as *providing* freedom. Undoubtedly, parents, like youth, also showed that supplies took over refrigerators and drawers within their homes and had to be toted everywhere they went. *“Every day and every time we go into the fridge we see RX,”* says a mother of a 15-year-old female describing a photo of insulin on a refrigerator shelf. Unlike youth, parents/caregivers also took many photos with narratives celebrating how grateful they were for specific supplies and conveyed that they made diabetes care easier and more manageable (Table 3). Roughly sixty percent of photo narratives about supplies by parents/caregivers were about the positive aspects of supplies and diabetes technology, whereas less than five percent of youth narratives were.

Another point of divergence in photo representations of youth and parents/caregivers was in images of encroachment: pictures that conveyed where diabetes trespassed on their lives (Table 4). For adolescents, encroachment was shown through images of their bodies – close-ups of finger tips, bruises from pump sites, images of subcutaneous injections with words like *“diabetes hurts,”* (13-year-old male) or *“this is where I constantly stab myself,”* (14-year-old female). For parents, encroachment was shown through images of clocks, alarms, and time to denote sleep disruption caused by night checks or the all-encompassing impact of diabetes care on daily schedules (Table 4). A mother of a 14-year-old son described a photo of an alarm with the words *“Normal alarm settings for years now include two night checks then @ 6:30 a.m., normal life still begins.”*

**Table 4:**
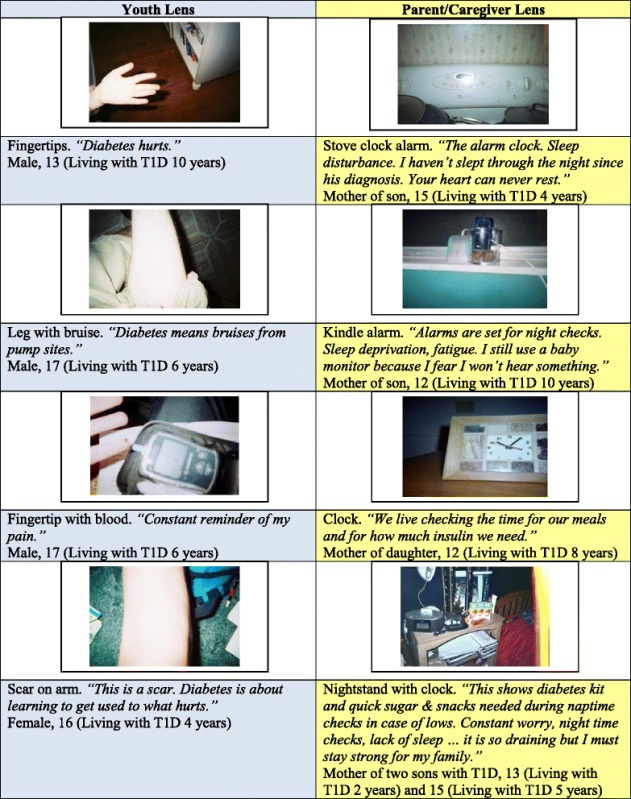
Areas of contrast: *Photos of encroachment*

## Conclusion

A content analysis of photos and narratives about what diabetes means to parents/caregivers of adolescents with T1D demonstrates several noteworthy areas of overlap and divergence with adolescent depictions. First, our data demonstrate that food-related negotiations and food-mindfulness surrounding diabetes care is denoted as a major source of contention by parents and youth. Second, the perception of diabetes supplies as *restricting* freedom by youth varies sharply from the portrayal of diabetes supplies as *providing* freedom by parents/caregivers and could serve as a trigger for diabetes conflicts within households. And third, the tendency for youth to show their bodies as a place of self-mutilation versus the parental tendency to show time/clocks and sleep disturbance could also signal an important area that may induce diabetes conflicts. For example, sleep fatigue, itself, limits one’s defenses when dealing with challenges associated with diabetes and puts parents in a particularly vulnerable state when faced with conflict. Moreover, these findings were consistent across income and educational thresholds signaling that such issues are paramount for families regardless of SES.

Our findings about food-specific challenges from the first study we conducted with adolescents have been published elsewhere [19] and indicate that this area of diabetes care warrants careful attention from providers. This follow-up study showed that parents/caregivers, too, devoted a significant portion of their photo-narratives to food issues. The role of dietitians is vital in helping families deal not only with medical nutrition therapy (MNT) but also to help prepare families for the tremendous social impact this part of diabetes care has on the entire family unit.

As for the findings related to diabetes supplies and areas of disease encroachment, there are important implications for glycemic control in T1D for adolescent populations. These data indicate that youth feel a loss of control over their bodies as well as their ability to prevent others around them (family and total strangers alike) from telling/asking/probing about their disease. Adolescence is a time where youth, regardless of T1D, desire greater autonomy and also strive towards the “norm of sameness” in peer culture [25–28]. Thus, while a new technology like a continuous glucose monitor (CGM) may be celebrated by a father for allowing him to know what his daughter’s blood glucose levels are while away from her, this simultaneously represents a limit to privacy for his daughter and could lead to maladaptive behaviors related to glycemic control (e.g. disabling a device, hiding/covering, etc.). It will be vital for adolescents with T1D to feel a sense of control and efficacy as much as possible when working out a shared system of how and when these technologies are used by parents.

The limitations of this study design include the underrepresentation of non-white participants and attrition (a 50 % return rate for completed packets). Also, there were not enough fathers/male caregivers in our sample to yield a systematic comparison of how maternal and paternal experiences may vary in diabetes. Future studies are needed to better elucidate the perspectives of teens who are non-white and of fathers/male caregivers specifically. However, despite these limitations, these findings provide new and unique insight into potential sources of diabetes-specific conflict within households. The photo-narratives reveal significant differences in how teens and parents/caregivers living with T1D perceive technologies like continuous glucose monitors and insulin pumps and allow a rare look at this disease through two sets of lenses that are equally important in considerations for clinical care. These findings also point to the importance of differential interventions aimed at reducing strain associated with T1D for teens and their parents: adolescents are most burdened by the intrusiveness of diabetes to their bodies whereas parents by the interruption to sleep and schedules. We present the methodology and techniques used in this research as a vehicle for providing improved communication and empathic role-taking within families as well as within clinical settings.

## Abbreviations

BGM: blood glucose monitoring
CGM: continuous glucose monitor
MNT: medical nutrition therapy
SES: socioeconomic status
T1D: type 1 diabetes

## References

[1] Laffel L, Connell A, Vangsness L, Goebel-Fabbri A, Mansfield A, Anderson B (2003). General quality of life in youth with type 1 diabetes. Diabetes Care.

[2] Drotar D, Ittenbach R, Rohan J, Gupta R, Pendley J, Delamater A (2013). Diabetes management and glycemic control in youth with type 1 diabetes: test of a predictive model. J Behav Med.

[3] Paris CA, Imperatore G, Klingensmith G, Petitti D, Rodriguez B, Anderson AM (2009). Predictors of insulin regimens and impact on outcomes in youth with type 1 diabetes: the SEARCH for Diabetes in Youth study. J Pediatr.

[4] Laffel L, Vangsness L, Connell A, Goebel-Fabbri A, Butler D, Anderson BJ (2003). Impact of ambulatory, family-focused teamwork intervention on glycemic control in youth with type 1 diabetes. J Pediatr.

[5] Haller MJ, Stavley MS, Silverstein JH (2004). Predictors of control of diabetes monitoring may be the key. J Pediatr.

[6] Wysocki T, Nansel TR, Holmbeck GN, Chen R, Laffel L, Anderson B (2009). Collaborative involvement of primary and secondary caregivers: associations with youths’ diabetes outcomes. J Pediatr Psychol.

[7] Dashiff C, Hardeman T, McLain R (2008). Parent–adolescent communication and diabetes: an integrative review. J Adv Nurs.

[8] Ingerski LM, Anderson BJ, Dolan LM, Hood KK (2010). Blood glucose monitoring and glycemic control in adolescence: contribution of diabetes-specific responsibility and family conflict. J Adolesc Health.

[9] Telo G, Volkening L, Butler D, Laffel L (2015). Salient characteristics of youth with type 1 diabetes initiating continuous glucose monitoring. Diabetes Technol Ther.

[10] Anderson B, Vangsness L, Connell A, Butler D, Goebel-Fabbri A, Laffel L (2002). Family conflict, adherence, and glycaemic control in youth with short duration type 1 diabetes. Diabet Med.

[11] Shorer M, David R, Schoenberg-Taz M (2011). Levavi-Lavii, Phillip M, Meyerovitch J. Role of parenting style in achieving metabolic control in adolescents with type 1 diabetes. Diabetes Care.

[12] Butler D, Zuehlke J, Tovar A, Volkening L, Anderson B, Laffel L (2008). The impact of modifiable family factors on glycemic control among youth with type 1 diabetes. Pediatr Diabetes.

[13] Wang C, Burris MA (1997). Photovoice: Concept, methodology, and use for participatory needs assessment. Health Educ Behav.

[14] Nykiforuk C, Vallianatos H, Nieuwendyk L (2011). Photovoice as a method for revealing community perceptions of the built and social environment. Int J Qual Methods.

[15] Findholt N, Michael Y, Davis M (2011). Photovoice engages rural youth in childhood obesity prevention. Public Health Nurs.

[16] Zenkov K, Harmon J (2009). Picturing a writing process: photovoice and teaching writing to urban youth. J Adolesc Adult Lit.

[17] Mitchell C, DeLange N, Moletsane R, Stuart J, Buthelezi T (2005). Giving a face to HIV and AIDS: on the uses of photo-voice by teachers and community health care workers working with youth in rural South Africa. Qual Res Psychol.

[18] Walker AF, Johnson C, Schatz DA, Silverstein JH, Lyles S, Rohrs HJ (2015). Using photography as a method to explore adolescent challenges and resilience in type 1 diabetes. Diabetes Spectr.

[19] Walker AF, Schatz DA, Silverstein JH, Parker KA, Aponick AU, Rohrs HJ (2013). Framing food and diabetes: exploring the perspectives of youth with type 1 diabetes through photography. Infant Child Adolesc Nutr.

[20] Walker AF, Johnson C, Schatz DA, Silverstein JH, Rohrs HJ (2015). Puppy love, adolescence, and chronic illness: the importance of pets for youth with type 1 diabetes. J Patient Exp.

[21] Krippendorff KH (2013). Content analysis: an introduction to its methodology.

[22] Riffe D, Lacy S, Fico FG (2005). Analyzing media messages: using quantitative content analysis in research.

[23] Glaser BG, Strauss AL (1967). The discovery of grounded theory.

[24] Strauss AL, Corbin J (1990). Basics of qualitative research: grounded theory procedures and research.

[25] Phinney JS, Kim-Jo T, Osorio S, Vilhjalmsdottir P (2005). Autonomy and relatedness in adolescent-parent disagreements: ethnic and developmental factors. J Adolesc Res.

[26] Hafen CA, Allen JP, Mikami AY, Gregory A, Hamre B, Pianta RC (2012). The pivotal role of adolescent autonomy in secondary school classrooms. J Youth Adolesc.

[27] McElhaney KB, Antonishak J, Allen JP (2008). “They like me, they like me not”: popularity and adolescents’ perceptions of acceptance predicting social functioning over time. Child Dev.

[28] Allen JP, Porter MR, McFarland FC (2005). The two faces of adolescents’ success with peers: adolescent popularity, social adaptation, and deviant behavior. Child Dev.

